# Importance of Quality of Life for Adherence to Sublingual Immunotherapy

**DOI:** 10.1155/2016/5186765

**Published:** 2016-07-18

**Authors:** Marie-Luise Lemberg, Peter Eberle, Kija Shah-Hosseini

**Affiliations:** ^1^Institute of Medical Statistics, Informatics and Epidemiology, University of Cologne, Lindenburger Allee 42, 50931 Cologne, Germany; ^2^Pediatric Practice for Pneumology/Allergology, Wilhelmshöher Allee 109, 34121 Kassel, Germany

## Abstract

*Background.* Nonperception of efficacy ranks among the most commonly cited causes for nonadherence to sublingual immunotherapy (SLIT). Quality of life (QoL) in patients is a determining factor influencing adherence. We investigated QoL and adherence separately in SLIT patients at one pediatric practice in Germany.* Methods.* We conducted a noninterventional, cross-sectional, retrospective, quality-of-life survey among pediatric patients treated with SLIT. QoL was assessed using the generic SF-12 health survey in German. The items contained in the SF-12 health survey are weighted, added up, and converted to obtain a physical component score (PCS) and a mental component score (MCS). Each component score ranges from 0 to 100; the higher the score, the better the QoL perceived.* Results*. 201 surveyed patients who had undergone SLIT showed PCS-12 of 49.3 (± 7.0) and MCS-12 of 52.6 (± 7.2). These figures correlate strongly with those reported for the German general population (*n* = 2453): PCS-12 of 49.6 (± 8.7) and MCS-12 of 52.3 (± 8.0). 70.2% (73) of 104 patients were adherent at this practice.* Conclusions*. QoL in the SLIT patients surveyed here appears as good as that of the general population. Adherence to SLIT at this practice was remarkably better than that reported elsewhere.

## 1. Introduction

Sublingual immunotherapy (SLIT) is the up-and-coming choice of therapy in hyposensitization. Especially in children, this therapy seems to be preferred because of its excellent safety profile and good tolerability [1]. Additionally, numerous studies have validated and verified the efficacy of SLIT [2]. Although SLIT is patient-managed and convenient, poor patient adherence has been reported, as for many other treatments of chronic conditions [3]. Specific data for adherence to SLIT vary widely [4], with dropout rates ranging from 49% to 82% of patients who do not follow the recommended three years of SLIT [3, 5]. Several postmarketing surveys and clinical trials have concluded that the causes for patients' nonadherence to SLIT are nonperception of efficacy, side effects, and costs [3, 4, 6]. An Italian study investigating the causes for nonadherence surveyed physicians who stated nonperception of efficacy as the most common motive for a patient to discontinue treatment [7, 8]. At the same time, other studies have proven the efficacy of the SLIT regimen [9, 10]. Finally, several studies have yielded conflicting results with respect to the problem of poor patient adherence to SLIT. An extensive American study disclosed the immense burden of allergic rhinitis in children and backed the proposition that, next to adverse events, a lack of therapy effectiveness is the most common reason for nonadherence in children and adult populations [11].

Studies offer a range of evidence on how strongly SLIT can enhance quality of life (QoL) by utilizing a disease-specific tool, the Rhinitis Quality of Life Questionnaire (RQLQ), to compare QoL before and after treatment [12]. Recently, studies have begun to explore the effect of SLIT on general QoL [2]. Since only little research on general QoL has been done so far, we conducted this generic health survey to acquire QoL data from SLIT patients at one practice and compare them to the data from the general population in Germany. Literature addressing nonadherence to SLIT is rare. Our objective was to evaluate the general QoL in a SLIT patient cohort and to prompt new thoughts on the reasons for nonadherence.

## 2. Methods

### 2.1. Participants

Patients treated with SLIT (carbamylated monomeric allergoid tablets, ®Lais tablets) from 2009 to 2014 at one pediatric practice (author P. Eberle) in Germany were selected for this cross-sectional, retrospective, quality-of-life survey. No clinical and demographical characteristics were documented to ensure the anonymity of the patients as required by the German data protection law. As long as personal data is not discernible, the data can be evaluated and analyzed for research purposes.

### 2.2. SF-12 Quality-of-Life Questionnaire

This survey implemented the generic SF-12 health survey in German to assess quality of life. The investigator (P. Eberle) sent the questionnaire to the parents of pediatric patients who had been prescribed SLIT from 2009 to 2014. Questionnaires were to be sent back to the investigator in a prepaid envelope provided to the patients' parents. Because no clinical and demographical characteristics were documented for reasons of data protection, the questionnaires could not be traced back to the respective patients. Valid questionnaires were subsequently analyzed. No follow-up was performed so that the questionnaires remained anonymous.

The questions contained in the SF-12 health survey are standard items which are weighted, added up, and converted to obtain a physical component score (PCS) and a mental component score (MCS). Each of the component scores ranges from 0 to 100; the higher the score, the better the QoL perceived [13].

The SF-12 is a standard measurement tool that evolved from the SF-36 in the USA and has been shown to be a reliable, comparable, and valid instrument for health assessment [14]. Evaluating the general health profile rather than disease-specific QoL allows a comparison across conditions and populations. Considering the results of the generic physical component summary scores (PCS-12) and mental component summary scores (MCS-12), our survey results can be compared to other generic SF-12 outcomes [15].

### 2.3. Adherence Data at the Same Setting

Patient adherence was to be evaluated separately for all patients at the participating pediatric practice who had completed a full 3-year treatment cycle as recommended in the WAO position paper for SLIT [10]. Hence, adherence was to be assessed for patients who had started immunization therapy between 2009 and 2011 and should theoretically have completed it between 2011 and 2014. We did not use the questionnaires but rather the patient data to analyze adherence. During this period, 104 patients started SLIT: 10 patients in 2009, 29 patients in 2010, and 65 patients in 2011. Dropouts were defined as patients who ceased to seek consultation at the practice before completing their 3-year treatment cycle.

## 3. Results

### 3.1. QoL

**Figure 1:**
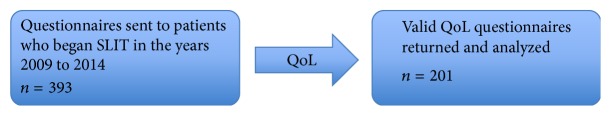
Patient flow for quality-of-life analysis.

The SF-12 questionnaire was sent to 393 eligible patients in April 2014. In total, 201 patients returned the completed SF-12 questionnaire. These 201 patients comprised the QoL population (Figure 1).

**Figure 2:**
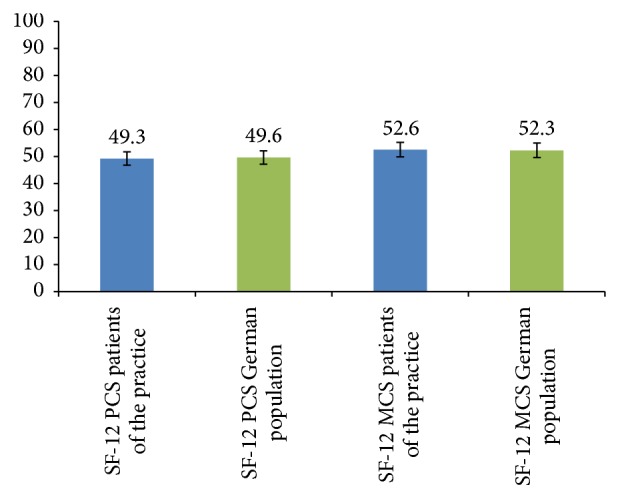
SF-12 results, blue: data from the practice, *n* = 201; SF-12 results, green: data from Gandek et al.'s study, *n* = 2453.

The analysis of the SF-12 health survey for the QoL population showed results that were practically equal to those reported for the German general population (Figure 2). PCS-12 of 49.28 (± 7.001) resulted for the QoL population, which is almost identical to that determined for the German general population. Similarly, the MCS-12 for the QoL population was 52.58 (± 7.232) and likewise almost the same as that of the German general population [14]. Calculations were performed using a standard procedure for SF-12 analysis [13].

### 3.2. Adherence

**Figure 3:**
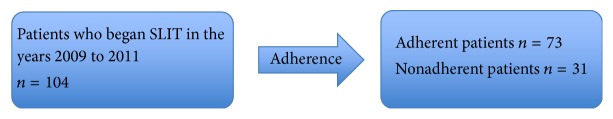
Patient flow for adherence analysis.

Overall, 104 patients began therapy between 2009 and 2011 (Figure 3). These patients were identified in a separate procedure based on anonymous raw patient data from the practice.

**Figure 4:**
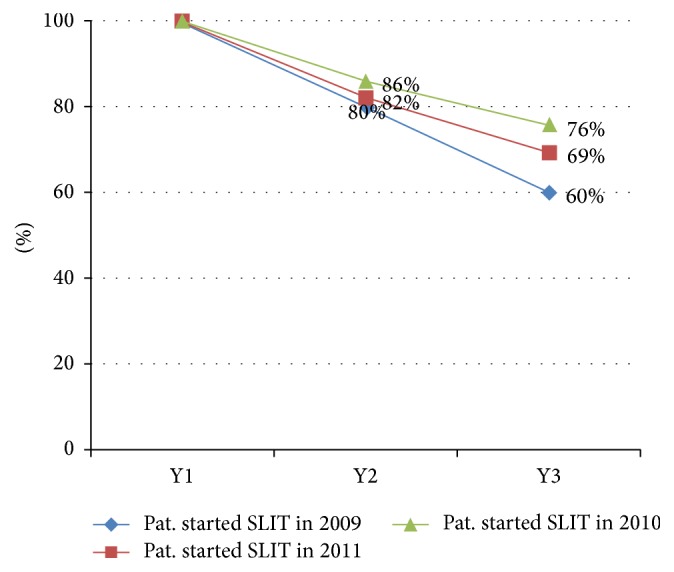
Adherence to SLIT in 104 patients who started immunotherapy in the years 2009 to 2011 at the participating practice.

In 2009, 10 patients started SLIT, 6 (60%) of whom finished the full 3-year therapy cycle. In 2010, 29 patients began SLIT and 22 of them (76%) completed the entire 3-year treatment schedule, yielding a dropout rate of 24%. In 2011, 65 patients started SLIT, 45 (69%) of whom completed the therapy. In total, 73 (70.2%) patients adhered to therapy and 31 patients dropped out prematurely. The dropouts were observed as a steady increasing number as shown in Figure 4.

## 4. Discussion

The patients responding to our survey who had been treated with SLIT assessed their QoL almost identically as did the German general population. To compare our QoL data, we used a study examining the health status of the general population of 9 European countries and extracted from this the general QoL in the German population between 18 and 74 years. The samples for this study were chosen representative for the general population in Germany according to sex, age, and regional distribution. The results of this nationwide survey showed PCS-12 of 49.3 (± 6.86) and MCS-12 of 52.4 (± 7.34). The patients participating in our survey who had been treated with SLIT had virtually the same mental and physical component scores as the German general population as stated in the abovementioned study from 1998 [14].

In connection with these similar scores, it is important to consider that QoL has been reported to be lower in patients suffering from allergies than in patients without them. Another study examined QoL in patients suffering from allergic rhinitis (AR) and patients suffering from AR and allergic asthma (AA) compared to a control group. Patients aged 20 to 44 years were observed, and the study did not differentiate between perennial and seasonal allergies, similar to a population study [16]. It used the generic SF-36 health survey, the outcomes of which are comparable with SF-12 results [14]. The results of this study of young adults revealed a significant decrease in the mental component scores of patients suffering from AR or AR and AA. Allergy patients showed a lower mental score (MCS-36 = 48.0) than the control group (MCS-36 = 51.3). Patients suffering from allergies also had a lower QoL than did the control group. The physical score PCS-36 in patients with AR and AA was 45.6 and 50.5 in the control group. Obviously, allergies compromise quality of life. Particularly, patients with both AR and AA suffer a significant loss of QoL in comparison to the control group [16].

SLIT appears to have a strong, positive impact on QoL in children suffering from allergies. QoL in children treated with SLIT at our practice is similarly good as that of the general German population. The present study therefore suggests that QoL in pediatric patients treated with SLIT might be comparable to that of the general population. It has been observed that untreated allergies compromise QoL. The QoL in patients with chronic conditions is dependent on the convenience, efficacy, and safety of the treatment used. These factors immediately affect QoL, which in turn directly influences adherence [17]. A general assessment such as the SF-12 permits a comparison between other conditions and the normal population, but its depth is limited [15]. QoL of the patients surveyed in our study was equal to that of the German general population. This finding suggests that patients treated with SLIT did perceive efficacy of the therapy. The results and conclusions drawn from our study may prompt researchers to reconsider the common opinion that nonperception of efficacy is one of the main causes for nonadherence to SLIT.

Patients who underwent SLIT in this practice were more compliant than patients in other studies [3, 5, 18]. The cumulative dropout rates between 24% and 40% in this particular practice were remarkably low; overall, 31 out of 104 patients (29.8%) dropped out and did not finish the therapy. Recent literature cites average dropout rates between 49% and 82% [3, 5]. One might assume that adherence to treatment increases when a patient perceives its efficacy [18].

One weakness of this study is the unknown overlap of data. The questionnaire was sent out to all patients who were prescribed SLIT from 2009 to 2014 (*n* = 393). Adherence data were retrieved from the same population—using patient data, not questionnaires—but only for patients beginning SLIT in the years 2009 to 2011 (*n* = 104). Questionnaires were not necessarily returned by all patients in the adherence population. The overlap, however, cannot be determined because the data were retrieved anonymously. Hence, we cannot analyze any subgroups.

As in many retrospective studies, the risk of recall bias is given in our study, too. In particular, the fact that parents answered the survey for their children makes it difficult to rule out recall bias. Still, in this case, recall bias through parents' reporting may be limited, because it was an anonymous survey about a treatment that is not explicitly exposed to public opinion and the survey was designed so as not to disclose any socially undesirable situation [19].

## 5. Conclusions

In summary, our results suggest that SLIT may have a positive impact on QoL. Adherence at this setting was remarkably good. Not only does efficacy of a therapy appear to be crucial for adherence, but also a holistic approach, the physician-patient relationship, and the quality of treatment delivery are determining factors [18]. Research has begun to develop strategies for enhancing patients' adherence to SLIT; extensive patient education as well as constant therapy monitoring can perceptibly increase adherence [3, 20]. The clinical benefit of a full cycle of SLIT can only be achieved with better adherence, which is dependent on a number of factors relating to the patient, the disease, the treatment itself, the physician, and the healthcare system in general [21]. Therefore, this up-and-coming route of therapy needs further research and guidelines on how optimal adherence can be achieved for SLIT patients. Our study gives an indication that adherence might be better if patients perceive good QoL.

## Abbreviations

AA: Allergic asthma
AR: Allergic rhinitis
MCS: Mental component score
PCS: Physical component score
QoL: Quality of life
RQLQ: Rhinitis Quality of Life Questionnaire
SLIT: Sublingual immunotherapy.
